# Nutritional and behavioral determinants of overweight and obesity among school-aged children in Shandong Province, China: evidence from a large-scale cross-sectional study

**DOI:** 10.3389/fnut.2026.1853192

**Published:** 2026-08-03

**Authors:** Wance Wang, Lei Yan, Bowen Xu, Xiangwei Pang, Qianqian Li, Ke Chen, Renkai Ge

**Affiliations:** 1 The School of Physical Education and Health, East China Jiaotong University, Nanchang, China; 2 School of Chemistry and Chemical Engineering, Qilu University of Technology (Shandong Academy of Sciences), Jinan, China; 3 Jafron Biomedical Co., Ltd., Jinan, China; 4 Shandong University of Art and Design, Jinan, China

**Keywords:** childhood obesity, lifestyle behaviors, overweight, risk factors, Shandong Province

## Abstract

**Background:**

Childhood overweight and obesity are pressing global health challenges with rising prevalence in China. Shandong Province, as a populous and economically developed region, provides a critical context for understanding regional patterns and determinants.

**Methods:**

A cross-sectional survey was conducted among 83,436 children aged 9–12 years from 155 schools in 16 cities (September–November 2025). Anthropometric measurements and validated questionnaires were used to assess weight status, demographics, family environment, and lifestyle factors. Overweight/obesity was classified using International Obesity Task Force (IOTF) cut-offs. Logistic regression identified associated risk and protective factors.

**Results:**

The prevalence of overweight/obesity was 19.0%. Rates were higher in boys than girls (19.9% vs. 18.2%, *p* < 0.001) and increased with age (11-year-olds 20.4% vs. 9-year-olds 18.0%, *p* < 0.001). Urban children had slightly higher prevalence than rural peers (19.6% vs. 18.7%, *p* = 0.002). Factors independently associated with overweight/obesity included parental overweight/obesity (OR = 1.18, 95% CI: 1.10–1.26), maternal gestational diabetes (OR = 1.53, 95% CI: 1.36–1.71), and high birth weight (OR = 1.16, 95% CI: 1.09–1.24). Lifestyle behaviors were strongly associated: <1 h/day physical activity increased risk, while >1.5 h/day was protective (OR = 0.83, 95% CI: 0.79–0.87); >8 h sleep reduced risk (OR = 0.66, 95% CI: 0.60–0.72); >3 h/day screen time (OR = 1.10, 95% CI: 1.02–1.20) and >3 h/day homework (OR = 1.49, 95% CI: 1.38–1.62) elevated risk; frequent fruit/vegetable intake was protective, whereas fast-food consumption >3 times/week increased risk (OR = 1.12, 95% CI: 1.05–1.20).

**Conclusion:**

Childhood overweight/obesity in Shandong Province is influenced by demographic, familial, and lifestyle factors. Findings highlight the importance of multilevel interventions—targeting family health behaviors, maternal metabolic management, school-based physical activity promotion, and community nutrition policies—to curb the rising burden of obesity.

## Introduction

Childhood overweight and obesity have emerged as one of the most pressing public health challenges of the 21st century. According to the World Health Organization (1), the prevalence of overweight and obesity among children and adolescents has continued to rise globally. In 2022, over 390 million individuals aged 5–19 years were overweight, including 160 million who were obese, while approximately 35 million children under the age of five were overweight (1). These figures highlight that childhood obesity has become a major global public health concern. This upward trend has been particularly pronounced in low and middle-income countries, in some cases outpacing that observed in high-income nations. Large-scale epidemiological studies published in The Lancet further highlight the severity of the global obesity burden, reporting that since 1980, the prevalence of childhood obesity has increased nearly eightfold (2, 3). If no effective measures are implemented, obesity is projected to become one of the leading contributors to the global disease burden in children by 2030 (4, 5). Collectively, these findings underscore that childhood obesity is not confined to individual countries or regions but represents a systemic challenge requiring coordinated global action.

The present study focused on children aged 9–12 years, as this period represents a critical transitional stage between childhood and early adolescence characterized by rapid physical growth and the initial formation of stable dietary and physical activity behaviors (6). During this stage, children begin to develop greater autonomy in food choices and lifestyle behaviors while still being strongly influenced by family and school environments (7, 8). Importantly, this age group precedes full pubertal development, making it a key window for early identification and prevention of overweight and obesity before long-term behavioral patterns become established (9).

In China, the world’s most populous developing country, the burden of childhood overweight and obesity has also become increasingly prominent. According to the Report on Nutrition and Chronic Disease Status of Chinese Residents (2020), the prevalence of overweight and obesity among children and adolescents aged 6–17 years reached 11.1 and 7.9%, respectively—representing two and three-fold increases compared with 2002 (10). National epidemiological data further indicate that the urban–rural disparity in childhood overweight and obesity is gradually narrowing, with the problem spreading to broader regions (11–13). Shandong Province, one of the most populous and economically developed areas of China, has consistently reported higher rates of childhood overweight and obesity than the national average (14, 15). This trend reflects both the general risk characteristics of the eastern coastal regions and the province’s unique demographic features, including its large population, pronounced urban–rural differences, and substantial educational pressures (16, 17). Therefore, large-scale and systematic epidemiological investigations are urgently needed to elucidate the prevalence patterns and key determinants of childhood obesity in Shandong, thereby providing robust evidence to inform region-specific and targeted interventions.

The adverse effects of childhood obesity on both physiological and psychological health have been well documented (18). Childhood obesity is associated with an increased risk of metabolic disorders, cardiovascular diseases, and adverse psychosocial outcomes that may persist into adulthood (19–23). Beyond its health consequences, accumulating evidence suggests that childhood overweight and obesity are influenced by a complex interplay of demographic, familial, behavioral, and environmental factors. Previous studies have identified sex, age, parental weight status, birth weight, dietary habits, physical activity, sedentary behavior, sleep duration, and socioeconomic characteristics as important factors associated with childhood obesity (24–26). In particular, unhealthy dietary patterns, insufficient physical activity, prolonged screen exposure, and inadequate sleep have consistently been linked to an increased likelihood of overweight and obesity among school-aged children. Family-related factors, including parental obesity, parental lifestyle habits, and maternal gestational diabetes, have also been recognized as important contributors to childhood weight status (27–29). However, the relative importance of these factors may differ across regions because of variations in socioeconomic development, educational pressures, urbanization, and lifestyle environments. Therefore, region-specific investigations are needed to better understand the determinants of childhood overweight and obesity and to support the development of targeted prevention strategies. However, evidence simultaneously examining nutritional, behavioral, and family-related determinants of childhood overweight and obesity remains limited in eastern coastal regions of China, particularly in large population-based samples.

In response to the growing burden of childhood overweight and obesity, the Chinese government has implemented a series of national strategies, including the “Healthy China 2030” initiative and subsequent obesity prevention programs, emphasizing healthy nutrition, physical activity promotion, and weight management among children and adolescents (30–32). These policies provide an important public health context for obesity prevention and control in China.

Despite increasing research on childhood obesity, evidence simultaneously examining nutritional, behavioral, and family-related determinants remains limited, particularly in eastern coastal regions of China. Furthermore, factors such as academic workload and maternal gestational diabetes have received relatively little attention in large population-based studies. Against this background, the present study focused on children aged 9–12 years in Shandong Province and used a large-scale cross-sectional survey to investigate the prevalence of overweight and obesity and their associations with nutritional, behavioral, and family-related factors. Specifically, dietary behaviors (fruit, vegetable, and fast-food consumption), lifestyle behaviors (physical activity, sleep duration, screen time, and homework time), parental characteristics, and maternal gestational diabetes were comprehensively evaluated. By integrating these multidimensional factors, this study aims to provide a more comprehensive understanding of childhood overweight and obesity and to generate evidence for targeted prevention strategies in Shandong Province and other eastern coastal regions of China.

## Materials and methods

### Study design and participants

This study was conducted in accordance with the Strengthening the Reporting of Observational Studies in Epidemiology (STROBE) guidelines (33). To comprehensively assess the prevalence of overweight and obesity among children aged 9–12 years in Shandong Province, a scientifically designed sampling procedure was implemented from September to November 2025. A total of 155 primary schools across all 16 prefecture-level cities in Shandong Province were included in the study, targeting children aged 9–12 years. To enhance the representativeness of the study population, a multistage stratified cluster sampling method was employed. First, within each city, primary schools were stratified according to their geographic location (urban and rural areas). Schools from both urban and rural settings were then selected with the assistance of local education authorities to ensure broad geographic coverage across the province. In the second stage, grade-based stratified sampling was conducted within each selected school, ensuring balanced representation across all age groups (9–12 years). Subsequently, cluster sampling was implemented by defining natural classes within each grade as sampling units, from which classes were randomly selected. All eligible students in the selected classes were invited to participate. This multistage sampling approach was designed to improve sample representativeness and enhance the reliability of the collected data.

A total of 83,436 children aged 9–12 years were ultimately included in the study. Exclusion criteria encompassed students with severe organ diseases, physical disabilities or deformities, and those who had used weight-influencing medications (e.g., hormonal drugs) within the past 6 months. All eligible participants completed anthropometric measurements and a structured questionnaire, achieving a 100% participation rate, with a valid response rate of 97.62%.

### Assessment of overweight/obesity and questionnaire survey

All participants and field investigators received standardized training to ensure the accuracy of height and weight measurements and the consistency of epidemiological data collection. Prior to the survey, the research team explained the study objectives, procedures, and potential implications to the students in age-appropriate language, ensuring that children could provide their assent to participate. Questionnaires were completed within one day through a child–parent collaborative approach. Considering the cognitive ability of children aged 9–12 years, the child-reported section was completed by children under the assistance of their parents or legal guardians, while the parent-reported section was completed independently by parents or legal guardians, particularly for items requiring objective information or medical history, such as maternal gestational diabetes. All questionnaires were completed anonymously, systematically collected, and promptly entered into a secure database. The study protocol was approved by the Ethics Committee of East China Jiaotong University (Approval No. 202510110001), and all procedures were conducted in accordance with relevant guidelines and regulations for research involving minors. Written informed consent was obtained from all legal guardians, while students provided written or verbal assent according to their age and comprehension, consistent with local ethical standards for research with children.

Prior to each assessment, all measurement instruments were calibrated according to the manufacturers’ instructions and underwent periodic verification throughout the data collection period. To ensure comparability of results across regions, all assessors received standardized training, including theoretical instruction and practical demonstrations, with particular emphasis on instrument operation, measurement procedures, and error minimization. Following training, personnel were required to pass a rigorous competency assessment. During data collection, senior researchers conducted regular supervision and quality control to maintain data integrity.

During anthropometric measurements, participants were instructed to empty their bladders and remove shoes, hats, and outer garments. They stood upright in the center of the scale platform with arms relaxed at their sides and eyes facing forward. Each child was measured three consecutive times, and the mean value was used for analysis. Height was recorded to the nearest 0.1 cm and weight to the nearest 0.1 kg. Body mass index (BMI) was calculated as weight (kg) divided by height squared (m^2^). Overweight and obesity were defined according to the sex and age-specific cut-off points proposed by the International Obesity Task Force, chosen to facilitate international comparability, though prevalence estimates may differ slightly from those based on Chinese national BMI references (34). To assess inter-rater reliability, approximately 10% of the sample was independently measured by different assessors, and intraclass correlation coefficients (ICCs) were calculated, all exceeding 0.89, indicating good to excellent measurement reliability.

The questionnaire assessed both demographic characteristics—including sex, age, residence, household income, parental education, parental weight status, parental physical activity, maternal gestational diabetes history, and birth weight—and lifestyle behaviors, such as daily physical activity, sleep duration, screen and homework time, and weekly intake frequency of meat, vegetables, fruits, eggs, milk, and fast food. Information on maternal gestational diabetes mellitus (GDM) was obtained through parent-reported questionnaires completed by the children’s parents or guardians. Respondents were asked whether the child’s mother had ever been diagnosed with gestational diabetes during pregnancy by a healthcare professional. The categorization of behavioral variables was based on a combination of international public health recommendations, previous epidemiological studies, and the distribution characteristics of the study population. For physical activity, categories (<60 min/day, 60–90 min/day, and >90 min/day) were defined with reference to the WHO recommendation that school-aged children engage in at least 60 min of daily moderate-to-vigorous physical activity (34, 35). Categories for sleep duration and dietary behaviors were established according to previous childhood obesity studies and the distribution of responses in the present sample to facilitate statistical analysis and interpretation (36–38). Prior to formal implementation, the questionnaire underwent reliability and validity evaluation. Test–retest reliability was assessed in a 10% subsample over a two-week interval, demonstrating good temporal stability. Content validity was independently evaluated by five experts with doctoral degrees, including two public health scholars, two physical education scholars, and one pediatric epidemiologist. Each item was rated on a 5-point Likert scale, and item-level content validity indices (I-CVI) and scale-level CVI (S-CVI) were calculated, with only items achieving I-CVI ≥ 0.80 retained. Items were further refined through consensus discussion to enhance clarity and cultural appropriateness.

In accordance with the STROBE statement, data integrity was rigorously assessed prior to statistical analysis. Questionnaires with more than 10% missing responses were excluded during the data cleaning process to ensure overall data quality. For the retained questionnaires, the proportion of missing values for key variables was less than 5%, and these missing values were handled using multiple imputation based on the Markov chain Monte Carlo (MCMC) method to minimize potential bias and maximize data utilization.

### Statistical analysis

A database was established using EpiData 3.1 and verified through double independent data entry. Statistical analyses were performed using SPSS version 27.0. Continuous variables are presented as mean ± standard deviation (SD), while categorical variables are described using frequencies and percentages [*n* (%)]. Prior to analysis, data were screened for completeness and consistency, and categorical variables were coded according to standardized criteria.

Associations between sociodemographic characteristics, family factors, lifestyle behaviors, and childhood overweight/obesity were initially examined using chi-square (*χ*^2^) tests. Variables that were statistically significant in the univariate analyses were subsequently entered into multivariable unconditional logistic regression models to identify factors independently associated with overweight/obesity. In addition, sex-stratified multivariable logistic regression analyses were conducted to explore potential heterogeneity in associations between boys and girls. To improve model calibration, separate multivariable logistic regression models were constructed independently for boys and girls, with candidate variables selected based on the results of the sex-specific univariate analyses. Variables that showed no statistically significant association with overweight/obesity in the sex-specific multivariable analyses were excluded from the final models to improve model parsimony and calibration. Prior to model fitting, multicollinearity among independent variables was assessed using variance inflation factors (VIF) and tolerance statistics. Results are presented as odds ratios (ORs) and corresponding 95% confidence intervals (CIs). Statistical significance was set at a two-sided *α* level of 0.05 (39). The Hosmer–Lemeshow test was employed to assess the goodness-of-fit and calibration of the final logistic regression model, ensuring the robustness of the analytical framework.

## Results

To identify key determinants of childhood overweight and obesity, both univariate and multivariate analyses were conducted on sociodemographic characteristics, behavioral patterns, and lifestyle factors. The objective was to determine variables significantly associated with overweight/obesity, thereby providing evidence to inform targeted health interventions and public health strategies.

Among the valid respondents, the sample demonstrated a balanced sex distribution, with 42,027 boys (50.37%) and 41,409 girls (49.63%). The age distribution was as follows: 17,279 9-year-olds (20.71%), 20,440 10-year-olds (24.50%), 21,938 11-year-olds (26.29%), and 23,779 12-year-olds (28.50%). The mean age of the study population was 10.63 ± 1.10 years.

### Univariate analysis of overweight and obesity by sociodemographic characteristics

The distribution of overweight and obesity across different sociodemographic groups was examined using chi-square (*χ*^2^) tests (Table 1). Significant sex differences were observed (*χ*^2^ = 40.014, *p* < 0.001), with boys exhibiting a higher prevalence of overweight/obesity (19.89%) compared to girls (18.23%). Age-stratified analysis also revealed significant differences (*χ*^2^ = 38.063, *p* < 0.001), with the highest prevalence observed among 11-year-olds (20.38%) and the lowest among 9-year-olds (18.01%), suggesting a gradual accumulation of weight-related issues in the middle to upper grades. Urban–rural disparities were statistically significant as well (*χ*^2^ = 9.803, *p* = 0.002), with urban children showing a slightly higher prevalence (19.57%) than their rural counterparts (18.70%).

**Table 1:** Distribution of overweight/obesity rates among participants with different demographic characteristics in Shandong Province, 2025.

Variables	Normal weight *n* (%)	Overweight/Obesity *n* (%)	*χ*2	*P*
Gender			40.014	<0.001
Boys	33,667 (80.11)	8,360 (19.89)		
Girls	33,884 (81.83)	7,525 (18.23)		
Age (years)			38.063	<0.001
9	19,496 (81.99)	4,283 (18.01)		
10	16,490 (80.68)	3,950 (19.32)		
11	13,758 (79.62)	3,521 (20.38)		
12	17,807 (81.17)	4,131 (18.83)		
Residence			9.803	0.002
Urban	25,959 (80.43)	6,318 (19.57)		
Rural	41,592 (81.30)	9,567 (18.70)		
Household annual income (CNY)			7.590	0.055
≤ 100,000	22,805 (81.39)	5,216 (18.61)		
100,001 ~ 200,000	30,372 (80.91)	7,167 (19.09)		
200,001 ~ 300,000	8,616 (80.61)	2072 (19.39)		
≥ 300,000	5,758 (80.11)	1,430 (19.89)		
Paternal education level			4.532	0.104
Junior high school or below	12,391 (81.52)	2,809 (18.48)		
Senior high school	19,871 (81.01)	4,657 (18.99)		
Above senior high school	35,289 (80.74)	8,419 (19.26)		
Maternal education level			2.029	0.363
Junior high school or below	14,143 (81.26)	3,262 (18.74)		
Senior high school	18,439 (81.07)	4,306 (18.93)		
Above senior high school	34,969 (80.79)	8,317 (19.21)		
Parental BMI status			32.454	<0.001
Neither overweight/obese	48,642 (81.41)	11,104 (18.59)		
One parent overweight/obese	14,631 (80.11)	3,633 (19.89)		
Both parents overweight/obese	4,277 (78.84)	1,148 (21.16)		
Whether parents liked physical activity			15.280	<0.001
Neither side of the parents liked	35,785 (80.51)	8,661 (19.49)		
One side of the parents liked	17,173 (81.16)	3,986 (18.84)		
Both sides of the parents liked	14,593 (81.84)	3,238 (18.16)		
Maternal gestational diabetes			49.248	<0.001
No	66,357 (81.09)	15,469 (18.91)		
Yes	1,194 (74.16)	416 (25.84)		
Birth weight			20.573	<0.001
Normal	57,954 (81.11)	13,500 (18.89)		
Low birth weight	5,132 (81.36)	1,176 (18.64)		
High birth weight	4,465 (78.69)	1,209 (21.31)		

With regard to family factors, no significant associations were observed between household income or parental education and childhood overweight/obesity (all *p* > 0.05), suggesting that economic status and educational attainment may have limited influence on weight outcomes in this age group. In contrast, parental weight status was significantly associated with children’s weight status (*χ*^2^ = 32.454, *p* < 0.001), with the highest prevalence observed among children whose both parents were overweight or obese (21.16%), highlighting the importance of genetic factors and shared family lifestyle. Similarly, parental physical activity showed significant differences (*χ*^2^ = 15.280, *p* < 0.001), with children from families in which both parents regularly exercised exhibiting the lowest prevalence of overweight/obesity (18.16%), indicating a protective effect of healthy lifestyle habits.

Maternal history of gestational diabetes was also significantly associated with childhood overweight/obesity (*χ*^2^ = 49.248, *p* < 0.001), with offspring of affected mothers showing a markedly higher prevalence (25.84%). Birth weight was similarly related to weight status (*χ*^2^ = 20.573, *p* < 0.001), with high birth weight children exhibiting the highest prevalence of overweight/obesity (21.31%) compared to those with normal or low birth weight.

### Univariate analysis of childhood overweight and obesity

This study further examined the associations between children’s daily lifestyle behaviors and overweight/obesity (Table 2). Physical activity was significantly associated with weight status (*χ*^2^ = 86.382, *p* < 0.001). Children engaging in less than 1 h of daily exercise had the highest prevalence of overweight/obesity (20.32%), whereas those exercising more than 1.5 h exhibited the lowest prevalence (17.44%), highlighting the protective role of regular physical activity in weight management. Sleep duration also showed a significant association (*χ*^2^ = 15.834, *p* < 0.001); children sleeping less than 6 h had a higher prevalence of overweight/obesity (19.95%) compared with those sleeping more than 8 h (18.23%), suggesting that insufficient sleep may increase obesity risk. Screen time was similarly related to overweight/obesity (*χ*^2^ = 31.598, *p* < 0.001). The prevalence was highest among children with more than 3 h of daily screen exposure (21.48%) and lowest among those with less than 1 h (18.58%), indicating the adverse impact of sedentary behaviors and excessive screen use on weight health. A comparable trend was observed for homework duration (*χ*^2^ = 18.178, *p* < 0.001), with children spending more than 3 h daily showing a higher prevalence of overweight/obesity (19.74%).

**Table 2:** Univariate analysis of overweight/obesity rates among study participants in Shandong Province, 2025.

Variables	Normal weight *n* (%)	Overweight/Obesity *n* (%)	*χ*2	*P*
Daily physical exercise time (hours)			86.382	<0.001
< 1	32,064 (79.68)	8,177 (20.32)		
1 ~ 1.5	19,844 (81.84)	4,403 (18.16)		
> 1.5	15,643 (82.56)	3,305 (17.44)		
Daily sleep duration (hours)			15.834	<0.001
< 6	4,956 (80.05)	1,235 (19.95)		
6 ~ 8	42,966 (80.70)	10,274 (19.30)		
> 8	19,629 (81.77)	4,376 (18.23)		
Daily screen time (hours)			31.598	<0.001
< 1	44,516 (81.42)	10,156 (18.58)		
1 ~ 3	19,121 (80.41)	4,658 (19.59)		
> 3	3,914 (78.52)	1,071 (21.48)		
Daily homework time (hours)			18.178	<0.001
< 1	32,341 (81.57)	7,309 (18.43)		
1 ~ 3	25,467 (80.47)	6,180 (19.53)		
> 3	9,743 (80.26)	2,396 (19.74)		
Number of times eating meat per week			3.279	0.194
< 3	31,963 (81.22)	7,390 (18.78)		
3 ~ 4	24,504 (80.75)	5,843 (19.25)		
> 4	11,084 (80.69)	2,652 (19.31)		
Number of times eating vegetables per week			17.059	<0.001
< 4	5,472 (79.69)	1,395 (20.31)		
4 ~ 5	16,644 (80.37)	4,065 (19.63)		
> 5	45,435 (81.34)	10,425 (18.66)		
Number of times consuming fruit per week			9.516	0.009
< 4	5,493 (79.99)	1,374 (20.01)		
4 ~ 5	16,677 (80.53)	4,032 (19.47)		
> 5	45,381 (81.24)	10,479 (18.76)		
Number of times eating eggs per week			5.947	0.051
< 4	8,209 (80.25)	2020 (19.75)		
4 ~ 5	41,185 (80.92)	9,711 (19.08)		
> 5	18,157 (81.38)	4,154 (18.62)		
Number of times drinking milk per week			3.221	0.200
< 4	10,184 (80.68)	2,439 (19.32)		
4 ~ 5	42,080 (80.87)	9,952 (19.13)		
> 5	15,287 (81.40)	3,494 (18.60)		
Number of times eating fried or high-calorie fast food per week			14.095	<0.001
< 2	49,097 (81.25)	11,328 (18.75)		
2 **~** 3	13,133 (80.43)	3,195 (19.57)		
> 3	5,321 (79.62)	1,362 (20.38)		

In terms of dietary behaviors, both vegetable (*χ*^2^ = 17.059, *p* < 0.001) and fruit intake frequency (*χ*^2^ = 9.516, *p* = 0.009) were significantly associated with overweight/obesity, with lower intake linked to higher prevalence. In contrast, consumption of meat, eggs, and milk showed no significant associations (*p* > 0.05), suggesting that these foods may not be decisive factors in this age group. However, frequent consumption of high-calorie fast food was strongly associated with overweight/obesity (*χ*^2^ = 14.095, *p* < 0.001); children consuming fast food more than three times per week had the highest prevalence (20.38%), underscoring the detrimental influence of unhealthy dietary habits.

### Multivariable logistic regression analysis of childhood overweight and obesity

To identify factors independently associated with childhood overweight/obesity, variables that were statistically significant in the univariate analyses were entered into a multivariable logistic regression model (Table 3). The results showed that, compared with boys, girls had significantly lower odds of overweight/obesity (OR = 0.896, 95% CI: 0.853–0.941, *p* < 0.001). Regarding age, using 9-year-olds as the reference group, children aged 10 years (OR = 1.091, 95% CI: 1.040–1.145, *p* < 0.001), 11 years (OR = 1.163, 95% CI: 1.107–1.222, *p* < 0.001), and 12 years (OR = 1.055, 95% CI: 1.006–1.107, *p* = 0.026) all exhibited significantly elevated risks of overweight/obesity. In terms of residential setting, rural children had a lower risk compared with their urban counterparts (OR = 0.943, 95% CI: 0.910–0.977, *p* = 0.001).

**Table 3:** Multivariate analysis of overweight/obesity rates among study participants in Shandong Province, 2025.

Variables	OR (95% CI)	*P*
Gender		
Boys	1	
Girls	0.896(0.853–0.941)	<0.001
Age (years)		
9	1	
10	1.091(1.040–1.145)	<0.001
11	1.163(1.107–1.222)	<0.001
12	1.055(1.006–1.107)	0.026
Residence		
Urban	1	
Rural	0.943(0.910–0.977)	0.001
Parental BMI status		
Neither overweight/obese	1	
One parent overweight/obese	1.087(1.042–1.133)	<0.001
Both parents overweight/obese	1.175(1.097–1.259)	<0.001
Whether parents liked physical activity		
Neither side of the parents liked	1	
One side of the parents liked	0.959(0.920–1.000)	0.050
Both sides of the parents liked	0.915(0.875–0.957)	<0.001
Maternal gestational diabetes		
No	1	
Yes	1.527(1.363–1.711)	0.001
Birth weight		
Normal	1	
Low birth weight	0.984(0.921–1.052)	0.644
High birth weight	1.162(1.087–1.242)	<0.001
Daily physical exercise time (hours)		
< 1	1	
1 ~ 1.5	0.870(0.835–0.906)	<0.001
> 1.5	0.829(0.793–0.867)	<0.001
Daily sleep duration (hours)		
< 6	1	
6 ~ 8	0.890(0.831–0.953)	<0.001
> 8	0.660(0.603–0.722)	<0.001
Daily screen time (hours)		
< 1	1	
1 ~ 3	0.983(0.932–1.037)	0.538
> 3	1.104(1.019–1.196)	0.015
Daily homework time (hours)		
< 1	1	
1 ~ 3	1.218(1.165–1.273)	<0.001
> 3	1.494(1.380–1.617)	<0.001
Number of times eating vegetables per week		
< 4	1	
4 ~ 5	0.958(0.895–1.025)	0.215
> 5	0.901(0.846–0.959)	<0.001
Number of times consuming fruit per week		
< 4	1	
4 ~ 5	0.966(0.902–1.035)	0.326
> 5	0.923(0.867–0.983)	0.013
Number of times eating fried or high-calorie fast food per week		
< 2	1	
2 ~ 3	1.067(1.021–1.115)	0.004
> 3	1.122(1.053–1.195)	<0.001

Hosmer–Lemeshow goodness-of-fit test: *p* = 0.655.

Among family-related factors, parental weight status was significantly associated with childhood overweight/obesity. Compared with children of parents without overweight/obesity, had higher odds of overweight/obesity (OR = 1.087, 95% CI: 1.042–1.133, *p* < 0.001), and the odds were further increased when both parents were overweight/obese (OR = 1.175, 95% CI: 1.097–1.259, *p* < 0.001). Parental physical activity was negatively associated with childhood overweight/obesity: children whose one parent exercised regularly had a slightly reduced risk (OR = 0.959, 95% CI: 0.920–1.000, *p* = 0.050), and the protective association was stronger when both parents exercised (OR = 0.915, 95% CI: 0.875–0.957, *p* < 0.001). Maternal history of gestational diabetes was independently associated with childhood overweight/obesity (OR = 1.527, 95% CI: 1.363–1.711, *p* < 0.001). Regarding birth weight, no significant association was observed for low birth weight compared with normal weight (OR = 0.984, 95% CI: 0.921–1.052, *p* = 0.644), whereas high birth weight was significantly associated with increased risk (OR = 1.162, 95% CI: 1.087–1.242, *p* < 0.001).

With respect to lifestyle factors, physical activity was significantly associated with lower odds of overweight/obesity. Compared with children exercising less than 1 h per day, those engaging in 1–1.5 h (OR = 0.870, 95% CI: 0.835–0.906, *p* < 0.001) and more than 1.5 h of daily exercise (OR = 0.829, 95% CI: 0.793–0.867, *p* < 0.001) had significantly lower odds of overweight/obesity. Sleep duration was inversely associated with the odds of overweight/obesity: relative to <6 h, both 6–8 h (OR = 0.890, 95% CI: 0.831–0.953, *p* < 0.001) and >8 h of sleep (OR = 0.660, 95% CI: 0.603–0.722, *p* < 0.001) were associated with lower odds of overweight/obesity. In contrast, excessive screen time was detrimental; children with >3 h of daily screen exposure exhibited a higher risk (OR = 1.104, 95% CI: 1.019–1.196, *p* = 0.015), whereas 1–3 h showed no significant association (OR = 0.983, 95% CI: 0.932–1.037, *p* = 0.538). Homework time was positively associated with overweight/obesity risk, with 1–3 h (OR = 1.218, 95% CI: 1.165–1.273, *p* < 0.001) and >3 h per day (OR = 1.494, 95% CI: 1.380–1.617, *p* < 0.001) both conferring significantly increased risks compared to <1 h.

### Sex-stratified logistic regression analysis

To further explore potential sex-specific differences in factors associated with overweight/obesity, sex-stratified multivariable logistic regression analyses were performed (Table 4). The Hosmer-Lemeshow goodness-of-fit test indicated satisfactory calibration for both sex-specific models, with *p* values of 0.618 for boys and 0.938 for girls.

**Table 4:** Sex-stratified multivariate analysis of overweight/obesity among study participants in Shandong Province, 2025.

Variables	Boys		Girls	
	OR (95% CI)	*P*	OR (95% CI)	*P*
Age (years)				
9	1		1	
10	0.886(0.824–0954)	0.001	0.986(0.919–1.058)	0.699
11	0.868(0.808–0.934)	<0.001	0.942(0.878–1.010)	0.092
12	0.819(0.762–0.880)	<0.001	0.898(0.838–0.962)	0.002
Residence				
Urban	1		1	
Rural	0.949 (0.901–0.998)	0.043	0.937 (0.892–0.984)	0.010
Parental BMI status				
Neither overweight/obese	1		1	
One parent overweight/obese	1.103(1.038–1.171)	0.001	1.073(1.013–1.138)	0.017
Both parents overweight/obese	1.157(1.046–1.278)	0.004	1.200(1.092–1.318)	<0.001
Whether parents liked physical activity				
Neither side of the parents liked	1		1	
One side of the parents liked	0.922(0.864–0.984)	0.014	0.945(0.888–1.006)	0.077
Both sides of the parents liked	0.482(0.391–0.594)	<0.001	0.724(0.587–0.893)	0.003
Maternal gestational diabetes				
No	1		1	
Yes	1.472(1.248–1.735)	<0.001	1.524(1.303–1.781)	<0.001
Birth weight				
Normal	1		1	
Low birth weight	0.955(0.868–1.052)	0.352	1.014(0.926–1.111)	0.764
High birth weight	1.217(1.107–1.338)	<0.001	1.109(1.010–1.218)	0.030
Daily physical exercise time (hours)				
< 1	1		1	
1 ~ 1.5	0.858(0.809–0.910)	<0.001	0.881(0.833–0.933)	<0.001
> 1.5	0.810(0.759–0.864)	<0.001	0.846(0.795–0.900)	<0.001
Daily sleep duration (hours)				
< 6	1		1	
6 ~ 8	0.894(0.810–0.986)	0.024	0.887(0.806–0.976)	0.014
> 8	0.665(0.584–0.757)	<0.001	0.656(0.578–0.744)	<0.001
Daily homework time (hours)				
< 1	1		1	
1 ~ 3	1.231(1.156–1.312)	<0.001	1.205(1.133–1.281)	<0.001
> 3	1.433(1.277–1.608)	<0.001	1.547(1.387–1.726)	<0.001
Number of times consuming fruit per week				
< 4	1		1	
4 ~ 5	1.023(0.925–1.131)	0.661	0.919(0.837–1.009)	0.077
> 5	0.952(0.868–1.045)	0.299	0.900(0.826–0.981)	0.017
Number of times drinking milk per week				
< 4	1		1	
4 ~ 5	1.001 (0.929–1.078)	0.986	1.031 (0.958–1.109)	0.415
> 5	1.800 (1.457–2.224)	<0.001	1.361 (1.101–1.683)	0.004

The Hosmer-Lemeshow test p-values were 0.618 for the boys subgroup and 0.938 for the girls.

Among boys, compared with those aged 9 years, boys aged 10, 11, and 12 years had significantly lower odds of overweight/obesity (*p* = 0.001, *p* < 0.001, and *p* < 0.001, respectively). Boys living in rural areas had slightly lower odds of overweight/obesity than those living in urban areas (OR = 0.949, 95% CI: 0.901–0.998). Having one or both parents with overweight/obesity was associated with increased odds of overweight/obesity, with a stronger association observed when both parents were overweight/obese. Boys whose one parent or both parents liked physical activity had significantly lower odds of overweight/obesity, with the strongest association observed when both parents liked physical activity (OR = 0.482, 95% CI: 0.391–0.594). Maternal gestational diabetes and high birth weight were associated with increased odds of overweight/obesity, whereas low birth weight was not significantly associated with overweight/obesity. Compared with boys engaging in less than 1 h/day of physical activity, those performing 1–1.5 h/day or more than 1.5 h/day had significantly lower odds of overweight/obesity. Similarly, sleeping 6–8 h/day or more than 8 h/day was associated with lower odds of overweight/obesity compared with sleeping less than 6 h/day. Longer homework duration was associated with higher odds of overweight/obesity. Fruit consumption was not significantly associated with overweight/obesity, whereas consuming milk more than five times per week was associated with increased odds of overweight/obesity (OR = 1.800, 95% CI: 1.457–2.224).

Among girls, compared with those aged 9 years, only those aged 12 years had significantly lower odds of overweight/obesity (OR = 0.898, 95% CI: 0.838–0.962). Girls living in rural areas also had lower odds of overweight/obesity than those living in urban areas (OR = 0.937, 95% CI: 0.892–0.984). Parental overweight/obesity, maternal gestational diabetes, high birth weight, longer homework duration, and consuming milk more than five times per week were positively associated with overweight/obesity. Girls whose both parents liked physical activity had lower odds of overweight/obesity (OR = 0.724, 95% CI: 0.587–0.893), whereas having only one parent who liked physical activity was not significantly associated with overweight/obesity. Compared with girls engaging in less than 1 h/day of physical activity, those performing 1–1.5 h/day or more than 1.5 h/day had significantly lower odds of overweight/obesity. Likewise, sleeping 6–8 h/day or more than 8 h/day was associated with lower odds of overweight/obesity. Consuming fruit more than five times per week was associated with lower odds of overweight/obesity (OR = 0.900, 95% CI: 0.826–0.981), whereas consuming fruit 4–5 times per week, low birth weight, and drinking milk 4–5 times per week were not significantly associated with overweight/obesity.

Overall, the associations between family-related factors and lifestyle behaviors with overweight/obesity were generally consistent between boys and girls. However, differences were observed in the magnitude and statistical significance of several variables, including age, residence, parental physical activity preference, fruit consumption, and milk consumption across the two sex-specific models.

## Discussion

This large-scale cross-sectional study of children aged 9–12 years in Shandong Province systematically assessed the prevalence and multifactorial determinants of overweight and obesity. The findings indicate that childhood overweight/obesity is associated with an interplay of demographic characteristics, family environment, and lifestyle behaviors. Specifically, sex, age, and urban–rural disparities were strongly associated with weight status; parental weight status, exercise habits, and maternal gestational diabetes were significantly associated with familial influences; while lifestyle factors—including physical activity, sleep duration, screen exposure, homework time, and dietary patterns—were important correlates. These results align with the life course perspective and the socio-ecological framework (40), providing empirical evidence to inform comprehensive strategies for the prevention and control of childhood obesity.

In this study, the overall prevalence of overweight/obesity among children aged 9–12 years was 19.04%, a level that can be considered moderately high compared with neighboring countries. Within China, the prevalence observed in Shandong was generally comparable to that reported in other economically developed eastern and coastal regions. For example, a large-scale survey conducted in Jiangsu Province among children aged 7–14 years reported a combined prevalence of overweight and obesity of approximately 26.9% (15.2% overweight and 11.7% obesity) (41). Similarly, studies from Shanghai have consistently identified childhood overweight and obesity as major public health concerns, with prevalence levels comparable to those observed in eastern coastal areas of China (42). Furthermore, national studies based on IOTF criteria have demonstrated that overweight and obesity are more prevalent in eastern and northeastern China than in western regions, reflecting the influence of rapid urbanization and socioeconomic development (43). These findings suggest that the prevalence observed in Shandong is not an isolated phenomenon but rather reflects a broader pattern of increasing childhood overweight and obesity in eastern China. For instance, data from the 2020 WHO COSI survey in Kazakhstan reported a prevalence of overweight and obesity of approximately 21.1 and 7.1%, respectively, among 8-year-old children (44). In Japan, multiple nationwide datasets indicate that the prevalence among school-aged children is generally lower than that observed in our study population, with estimates typically ranging between 10 and 12%, although showing a gradual upward trend (45). In Korea, the prevalence of childhood obesity increased from approximately 6.8% in 1998 to 10.0% in 2013, also lower or slightly lower than the rates reported in Shandong (46). A systematic review from India and South Asia revealed substantial regional heterogeneity, with urban areas showing pronounced increases; pooled estimates suggested prevalence rates of approximately 12.4% for overweight and 8.4% for obesity (47). These differences may be partly attributable to variations in diagnostic criteria, age stratification, and urban–rural sampling, as well as to cross-country differences in dietary patterns, opportunities for physical activity, and academic pressures (48, 49). Moreover, differences in the timing of surveys further complicate direct comparisons across studies.

With respect to demographic characteristics, boys exhibited a significantly higher risk of overweight/obesity compared with girls, a finding consistent with both national and international studies. Potential mechanisms may involve physiological as well as behavioral factors. Boys differ from girls in energy metabolism, endocrine regulation (e.g., androgen activity), and fat distribution patterns, making them more prone to visceral adiposity (50, 51). From a behavioral perspective, boys often display stronger preferences for energy-dense foods (52, 53); although they may participate more frequently in physical activity, this advantage can be offset by higher sedentary time and greater use of electronic devices (54, 55). Increasing age was also closely associated with a higher risk of overweight/obesity, which may reflect the cumulative effects of greater academic burden, reduced opportunities for physical activity, and increased dietary autonomy during school years (56, 57). Particularly in the 11–12 age range, children are transitioning into puberty, when alterations in growth hormone secretion, insulin sensitivity, and basal metabolic rate may further predispose them to excess weight gain (58, 59). Interestingly, although increasing age was associated with a higher risk of overweight/obesity in the overall analysis, the sex-stratified analyses revealed an inverse association among boys and a significant inverse association only among 12-year-old girls. This discrepancy suggests that the relationship between age and overweight/obesity may be modified by sex-specific developmental characteristics. During puberty, boys experience rapid increases in lean body mass and skeletal muscle under the influence of androgen secretion, resulting in higher resting energy expenditure (60, 61). Because BMI does not distinguish between fat mass and fat-free mass, the rapid accumulation of muscle mass may partially influence BMI-based weight classification in boys and contribute to the observed age-related differences. In contrast, girls undergo estrogen-mediated changes in body composition, with a greater tendency toward fat redistribution during pubertal maturation (62, 63). Furthermore, health-related behaviors also evolve with age during adolescence, and older children may gradually develop greater awareness of body weight and healthier lifestyle habits, particularly among girls (64, 65). These physiological and behavioral differences may partly explain why the age-related associations differed between the overall and sex-stratified analyses. Interestingly, our findings revealed that rural children had a slightly lower risk of overweight/obesity compared with their urban counterparts. This disparity may be associated with lifestyle differences, as urban children may experience higher energy intake, more sedentary behaviors, and greater academic pressures, compounded by easier access to energy-dense diets (66–68). These results highlight the need for targeted prevention strategies, with particular emphasis on promoting healthy lifestyle interventions among urban children.

The influence of family environment and early-life factors on childhood weight was also pronounced. Parental weight status was closely associated with children’s risk of overweight/obesity, reflecting both genetic susceptibility and the modeling effect of family lifestyle (69, 70). Regular parental physical activity not only promotes overall family health behaviors but also increases children’s opportunities for exercise and social support, and was associated with greater engagement in children’s physical activity enhancing their engagement in physical activity (71, 72). The observed positive association between maternal gestational diabetes and childhood overweight/obesity further supports the life course concept of “early programming (73)”: hyperglycemia during pregnancy can alter fetal pancreatic *β*-cell function and adipogenesis pathways, potentially exerting long-term effects on offspring weight through epigenetic mechanisms (74, 75). In addition, high birth weight was significantly associated with increased risk of overweight/obesity, potentially mediated by excessive fetal nutrition leading to higher adipocyte numbers, dysregulation of basal metabolic processes, and the cumulative effects of rapid early growth trajectories (76, 77). In contrast, low birth weight was not significantly associated with subsequent overweight/obesity in this population.

With regard to lifestyle factors, this study confirmed that regular physical activity and adequate sleep exert protective effects against childhood overweight and obesity. Consistent exercise is associated with higher energy expenditure, enhances muscle mass and basal metabolic rate, and positively modulates insulin sensitivity and inflammatory status (78, 79). Insufficient sleep has been associated with obesity, possibly through by altering ghrelin and leptin secretion, increasing nocturnal energy intake, and reducing overall physical activity (80, 81). Excessive screen time and longer homework duration were both associated with elevated overweight/obesity risk, reflecting a “high cognitive load–low energy expenditure” composite effect (82). Notably, while longer homework time appears to be a potential risk factor, its independent effect may be partly confounded by socioeconomic status, academic performance, or urban versus rural residency, suggesting that the observed association should be interpreted in the context of these underlying factors. In terms of dietary behaviors, frequent consumption of fruits and vegetables was associated with lower overweight/obesity risk, whereas frequent intake of energy-dense was positively associated with overweight/obesity, consistent with the established role of diet quality in energy balance regulation (83). Interestingly, intake of protein-rich foods such as meat, eggs, and dairy products was not significantly associated with overweight or obesity in this population. This finding aligns with several prior studies suggesting that moderate consumption of these foods may not contribute to excess weight in children and could support overall nutritional adequacy (84–86). It is possible that portion sizes, preparation methods, and overall dietary patterns modulate their impact on energy balance, highlighting the need for more detailed assessment of protein sources and nutrient composition in future research (87).

Despite providing valuable insights into the prevention and control of childhood overweight and obesity, this study has several limitations. First, the cross-sectional design precludes causal inference. Second, lifestyle variables were primarily based on self or parent-reported data, which may be subject to recall bias. Moreover, physical activity was assessed based on duration rather than activity intensity or energy expenditure (e.g., METs), which may have led to some degree of measurement bias. Additionally, information regarding maternal gestational diabetes mellitus (GDM) was obtained through parental self-report rather than medical record verification. Although GDM is a clinically significant pregnancy-related condition that is generally well remembered by mothers, recall bias and potential misclassification cannot be completely excluded. Therefore, the observed association between maternal GDM and childhood overweight/obesity should be interpreted with caution. Third, potential confounding factors, such as total energy intake, portion sizes, family dietary environment, and COVID-19-related changes in physical activity and screen time, were not assessed, which may have influenced the observed associations. Fourth, dietary assessment was limited to consumption frequency and did not capture portion sizes or total energy intake. Future research could improve dietary measurement by using more structured questionnaires that explicitly assess high-salt and high-sugar foods, sugar-sweetened beverages, and overall energy intake, allowing a more precise evaluation of dietary contributions to overweight and obesity. In addition, although sex-stratified analyses were conducted in the present study, age-specific stratified analyses were not further explored due to the relatively narrow age range (9–12 years). Future studies with broader age ranges are encouraged to examine potential age-related heterogeneity in associations with childhood overweight and obesity. Finally, the study was conducted exclusively in Shandong Province, which may limit the generalizability of the findings. Longitudinal cohort designs, combined with objective measurement tools (e.g., accelerometers) and mediation analyses, could help clarify causal pathways and provide stronger evidence to guide interventions for childhood obesity.

Childhood overweight and obesity have emerged as prominent public health challenges worldwide, and children in China, including those in Shandong Province, are similarly affected. The long-term physical and psychological consequences of obesity underscore the urgent need for large-scale, region-specific studies to provide robust scientific evidence for policy development and health interventions. This study helps to enrich the existing epidemiological evidence on childhood obesity in Shandong Province by providing updated regional data and identifying associated demographic, familial, and lifestyle factors. Furthermore, these findings have important implications for promoting healthy growth in children and advancing sustainable societal development. By generating evidence-based, child-centered interventions, this research contributes to fostering a generation with enhanced health literacy and supporting the sustainable construction of a healthier society.

## Conclusion

Based on a large-scale survey of children aged 9–12 years in Shandong Province, this study identified demographic, family, and lifestyle factors influencing overweight and obesity. Boys, older children, and urban residents were at higher risk, while healthy behaviors such as regular physical activity, adequate sleep, and balanced diet were protective. These findings suggest that childhood overweight and obesity are influenced by multiple interacting factors at the individual, family, and environmental levels. Therefore, comprehensive prevention efforts involving families, schools, and broader social environments may be warranted. These findings provide evidence to guide targeted interventions and public health policies to reduce childhood overweight and obesity.

## Data Availability

The original contributions presented in the study are included in the article/Supplementary material, further inquiries can be directed to the corresponding author.
